# Explainable machine learning to predict long-term mortality in critically ill ventilated patients: a retrospective study in central Taiwan

**DOI:** 10.1186/s12911-022-01817-6

**Published:** 2022-03-25

**Authors:** Ming-Cheng Chan, Kai-Chih Pai, Shao-An Su, Min-Shian Wang, Chieh-Liang Wu, Wen-Cheng Chao

**Affiliations:** 1 Division of Critical Care and Respiratory Therapy, Department of Internal Medicine, Taichung Veterans General Hospital, Taichung, Taiwan; 2 College of Science, Tunghai University, Taichung, Taiwan; 3 College of Engineering, Tunghai University, Taichung, Taiwan; 4 Artificial Intelligence Center, Tunghai University, Taichung, Taiwan; 5 Artificial Intelligence Studio, Taichung Veterans General Hospital, Taichung, Taiwan; 6 Department of Critical Care Medicine, Taichung Veterans General Hospital, Taichung, Taiwan; 7 Department of Industrial Engineering and Enterprise Information, Tunghai University, Taichung, Taiwan; 8 College of Medicine, Chung Hsing University, Taichung, Taiwan; 9 Department of Automatic Control Engineering, Feng Chia University, Taichung, Taiwan; 10 Big Data Center, National Chung Hsing University, Taichung, Taiwan

**Keywords:** Mortality prediction, Critical illness, Mechanical ventilation, Machine learning, Interpretability

## Abstract

**Background:**

Machine learning (ML) model is increasingly used to predict short-term outcome in critically ill patients, but the study for long-term outcome is sparse. We used explainable ML approach to establish 30-day, 90-day and 1-year mortality prediction model in critically ill ventilated patients.

**Methods:**

We retrospectively included patients who were admitted to intensive care units during 2015–2018 at a tertiary hospital in central Taiwan and linked with the Taiwanese nationwide death registration data. Three ML models, including extreme gradient boosting (XGBoost), random forest (RF) and logistic regression (LR), were used to establish mortality prediction model. Furthermore, we used feature importance, Shapley Additive exPlanations (SHAP) plot, partial dependence plot (PDP), and local interpretable model-agnostic explanations (LIME) to explain the established model.

**Results:**

We enrolled 6994 patients and found the accuracy was similar among the three ML models, and the area under the curve value of using XGBoost to predict 30-day, 90-day and 1-year mortality were 0.858, 0.839 and 0.816, respectively. The calibration curve and decision curve analysis further demonstrated accuracy and applicability of models. SHAP summary plot and PDP plot illustrated the discriminative point of APACHE (acute physiology and chronic health exam) II score, haemoglobin and albumin to predict 1-year mortality. The application of LIME and SHAP force plots quantified the probability of 1-year mortality and algorithm of key features at individual patient level.

**Conclusions:**

We used an explainable ML approach, mainly XGBoost, SHAP and LIME plots to establish an explainable 1-year mortality prediction ML model in critically ill ventilated patients.

**Supplementary Information:**

The online version contains supplementary material available at 10.1186/s12911-022-01817-6.

## Background

The long-term outcome is currently an emerging issue in critically ill patients due to increasing awareness of sequelae result from acute illness including coronavirus disease 2019 (COVID-19) infection [1–3]. Shankar-Hari et al. conducted a meta-analysis with 43 studies to address the 1-year mortality in critically ill septic patients discharged from intensive care unit (ICU) and found that the post-ICU 1-year mortality was approximately 16% [1]. Similarly, the COVID-ICU study group recently reported a number of sequelae after ICU discharge, and the mortality gradually reached approximately 31% (1298/4244) at 90 days after ICU admission for COVID-19 infection [3]. A number of recent studies, including our study focusing on the long-term impact of early fluid balance on 1-year mortality in critically ill cancer patients, have identified factors associated with the long-term mortality in critically ill patients [4, 5]. These evidence highlight the essential need to explore early determinants and early risk stratification for long-term outcome in critically ill patients.

Artificial intelligence (AI) is increasingly used in a wide range of fields, but the black-box issue remains the major concern for the application of AI in the medical field, particularly in critical care medicine, given decision support system without rationale is somehow imprudent for the physician [6–8]. Recently, explainable AI, including our recently published studies using explainable machine learning (ML) model in patients with severe influenza infection and critically ill ventilated patients, have been increasingly applied to interpret the AI model based on the domain knowledge and post-hoc analyses; therefore, the black-box issue can be mitigated through explainable ML approach [9–12].

In the present study, we linked the critical care database of Taichung Veterans General Hospital (TCVGH) with the death registration data of the National Health Insurance Research Database (NHIRD) in Taiwan to establish a critical care database containing data regarding long-term mortality. We employed the explainable ML approach to establish a long-term outcome prediction model and to address the distinct determinants for short-term and long-term mortality in critically ill ventilated patients.

## Materials and methods

### Ethical approval

The Institutional Review Board of Taichung Veterans General Hospital has approved the present study (TCVGH: CE20249B). The data were retrieved from the electronic medical record (EMR) at TCVGH, and informed consent was waived due to that the data were anonymised before analyses.

### Establishment of database

In the present study, the critical care database was established through retrieving data from the data warehouse at TCVGH, a tertiary care referral hospital with 1530 beds and six ICUs in central Taiwan. Patients who admitted to ICUs between 2015-July and 2018-July were included in this study, and we used the first ICU admission as the index ICU admission. The date-of-death was obtained from the death registration data of Taiwanese NHIRD, a single-payer and compulsory health insurance program with 99.9% coverage of the Taiwanese population in 2019, and the date-of-death should hence be accurate [13]. The dataset mainly consisted of the five clinical domains, including ventilation domain, fluid domain, physiology domain, lab domain, and severity score.

### Machine learning models

We employed three machine learning (ML) models, including Extreme gradient boosting (XGBoost), Random forest (RF), and Logistic regression (LR). In the setting of the key hyperparameters, the optimal values were determined by a grid search on potential value combinations of the parameters. Notably, we provided visualised explanation at domain-, feature- and individual levels to mitigate the issue of black-box of ML models. For the interpretability of the ML models at the domain level, we quantified the cumulative feature importance of the aforementioned clinical domains. In this study, the score of feature importance was quantified by the average gain across all splits of a feature used during the construction of the tree-based model. We then used SHAP and PDP for further visualise explanation at the feature level [14]. In brief, the SHAP summary plot was used to illustrate the strength as well as the direction of associations between key features and 30-day, 90-day and 1-year mortality, and the partial dependence plot (PDP) was used to show the marginal effect of features on the predicted outcome of key features. For further explanation at the individual level, we used LIME and SHAP force plots to illustrate the impact of key features at the individual level [15]. In brief, LIME gives an explanation of a classifier through approximating the key features by applying a locally linear model. The exported output of LIME was the explanation which represent the contribution of key features to the predicted outcome in an individual patient.

### Statistical analysis

The continuous data were presented as means ± standard deviations, and categorical data were expressed as frequencies (percentages). Fisher’s exact test and Student’s t-test and were used to measure the difference between the two groups. We divided the data into training dataset (80%) and testing dataset (20%) (see Additional file 1: Fig. S1 for the flow diagram of the study). We used the receiver operating characteristic (ROC) curve analysis, calibration curve and decision curve analysis to determine discrimination, accuracy and applicability of the predictive ML models in the testing sets [16, 17]. Furthermore, we used DeLong’s test to determine the difference between two area under curves (AUCs) [18]. Python version 3.6 was applied in the present study.

## Results

### Demographic and ventilatory data

**Fig. 1:**
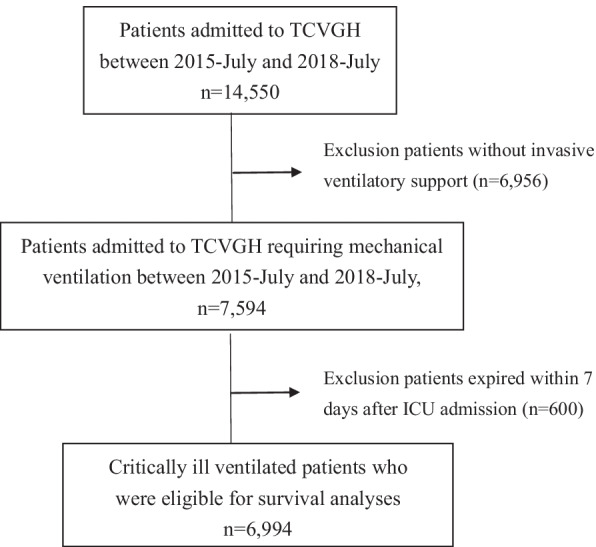
Flowchart of subject enrollment. *TCVGH* Taichung Veterans General Hospital, *ICU* intensive care unit

A total of 6994 critically ill patients requiring mechanical ventilation were enrolled, and 160 features were used in the present study (Fig. 1). The mean age of them was 64.1 ± 16.2 years, and 65.1% of enrolled subjects were male. We found that 34.4% (2405/6994) of patients died within 1 year after the index ICU admission. The non-survivors were more likely to be discharged from the medical ICU (50.0% vs. 18.8%, *p* < 0.01), to have a higher APACHE II score (26.3 ± 6.4 vs. 21.6 ± 6.6, *p* < 0.01), white blood cell counts (12,464.7 ± 14,292.0 vs. 11,414.5 ± 4310.7, *p* < 0.01), level of blood urea nitrogen (37.5 ± 30.4 vs. 24.0 ± 20. 7, *p* < 0.01), level of creatinine (1.9 ± 2.0 vs. 1.5 ± 1.8, *p* < 0.01), whereas a lower level of haemoglobin (9.9 ± 1.8 vs. 10.9 ± 1.9, *p* < 0.01) and albumin (3.0 ± 0.9 vs. 3.4 ± 0.8, *p* < 0.01) compared with those in the survivor group (Table 1). Table 2 summarises the main ventilatory parameters, and we found that patients in the non-survivor group tended to have a higher FiO2, PEEP and Ppeak than those in the survivor group (Table 2).

**Table 1:** Characteristics of the 6994 critically ill ventilated patients categorised by 1-year mortality

	All	Survivor	Non-survivor	*p* value
	N = 6994	N = 4589	N = 2405	
Demographic data				
Age (years)	64.1 ± 16.2	61.6 ± 16.1	68.7 ± 15.3	< 0.01
Sex (male)	4550 (65.1%)	2919 (63.6%)	1631 (67.8%)	< 0.01
Body mass index	24.3 ± 4.7	24.8 ± 4.7	23.3 ± 4.6	< 0.01
CCI categories				< 0.01
CCI: 0	960 (13.7%)	863 (18.8%)	97 (4.0%)	
CCI: 1–2	4054 (58.0%)	2700 (58.8%)	1354 (56.3%)	
CCI ≧ 3	1980 (28.3%)	1026 (22.4%)	954 (39.7%)	
ICU types				< 0.01
Medical ICU	2367 (33.8%)	1165 (25.4%)	1202 (50.0%)	
Surgical ICU	1480 (21.2%)	863 (18.8%)	617 (25.7%)	
Cardiac ICU	1441 (20.6%)	1209 (26.4%)	232 (9.6%)	
Neurological ICU	1706 (24.4%)	1352 (29.4%)	354 (14.7%)	
APACHE II	23.3 ± 6.9	21.6 ± 6.6	26.3 ± 6.4	< 0.01
Laboratory data (day-1)				
White blood cell count (count/μL)	11,775.6 ± 9091.8	11,414.5 ± 4310.7	12,464.7 ± 14,292.0	< 0.01
Hemoglobin (g/dL)	10.5 ± 1.9	10.9 ± 1.9	9.9 ± 1.8	< 0.01
Platelet (10^3^/μL)	185.40 ± 95.5	192.9 ± 90.9	170.9 ± 102.0	< 0.01
Albumin (mg/dL)	3.3 ± 0.8	3.4 ± 0.8	3.0 ± 0.9	< 0.01
BUN (mg/dL)	28.6 ± 25.3	24.0 ± 20.7	37.5 ± 30.4	< 0.01
Creatinine (mg/dL)	1.6 ± 1.9	1.5 ± 1.8	1.9 ± 2.0	< 0.01
Lactate (mg/dL)	16.0 ± 15.4	14.0 ± 13.4	19.9 ± 17.9	< 0.01
Outcome				
ICU-stay (day)	11.3 ± 10.7	9.2 ± 9.0	15.3 ± 12.4	< 0.01
Ventilator-day	8.6 ± 10.6	6.3 ± 8.6	13.0 ± 12.5	< 0.01
Hospital-stay (day)	27.3 ± 24.4	24.2 ± 23.5	33.3 ± 25.1	< 0.01

Data were presented as mean ± standard deviation and number (percentage)

*CCI* Charlson comorbidity index, *ICU* intensive care unit, *APACHE II* acute physiology and chronic health evaluation II, *BUN* blood urea nitrogen

**Table 2:** Respiratory parameters of critically ill ventilated subjects categorised by 1-year mortality

	All	Survivor	Non-survivor	*p* value
	N = 6994	N = 4589	N = 2405	
Day 1				
FiO_2_ (%)	47.1 ± 13.6	45.5 ± 12.2	50.2 ± 15.5	< 0.01
PEEP	5.9 ± 2.3	5.7 ± 2.2	6.3 ± 2.4	< 0.01
V_T_/PBW	8.7 ± 2.1	8.7 ± 2.0	8.8 ± 2.2	0.03
P_peak_	22.8 ± 5.0	22.2 ± 4.8	23.9 ± 5.2	< 0.01
Day 2				
FiO_2_ (%)	41.6 ± 8.9	41.1 ± 8.1	42.6 ± 10.1	< 0.01
PEEP	6.1 ± 2.5	5.8 ± 2.4	6.7 ± 2.7	< 0.01
V_T_/PBW	41.6 ± 8.9	41.1 ± 8.1	42.6 ± 10.1	< 0.01
P_peak_	22.4 ± 5.2	21.7 ± 4.9	23.8 ± 5.4	< 0.01
Day 3				
FiO_2_ (%)	40.4 ± 8.4	40.1 ± 7.8	41.0 ± 9.3	< 0.01
PEEP	6.1 ± 2.4	5.7 ± 2.2	6.6 ± 2.7	< 0.01
V_T_/PBW	8.7 ± 2.3	8.6 ± 2.2	8.8 ± 2.4	< 0.01
P_peak_	22.1 ± 5.3	21.4 ± 5.0	23.5 ± 5.6	< 0.01
Day 7				
FiO_2_ (%)	39.3 ± 8.4	39.0 ± 7.5	39.7 ± 9.8	< 0.01
PEEP	5.8 ± 2.0	5.5 ± 1.6	6.3 ± 2.4	< 0.01
V_T_/PBW	8.7 ± 2.4	8.6 ± 2.3	8.8 ± 2.5	< 0.01
P_peak_	20.9 ± 5.3	20.2 ± 4.8	22.4 ± 6.0	< 0.01

Data were presented as mean ± standard deviation

*PEEP* positive end-expiratory pressure, *V*_*T*_ tidal volume, *PBW* predicted body weight, *P*_*peak*_ peak pressure

### Comparisons among XGBoost, RF and LR

**Fig. 2:**
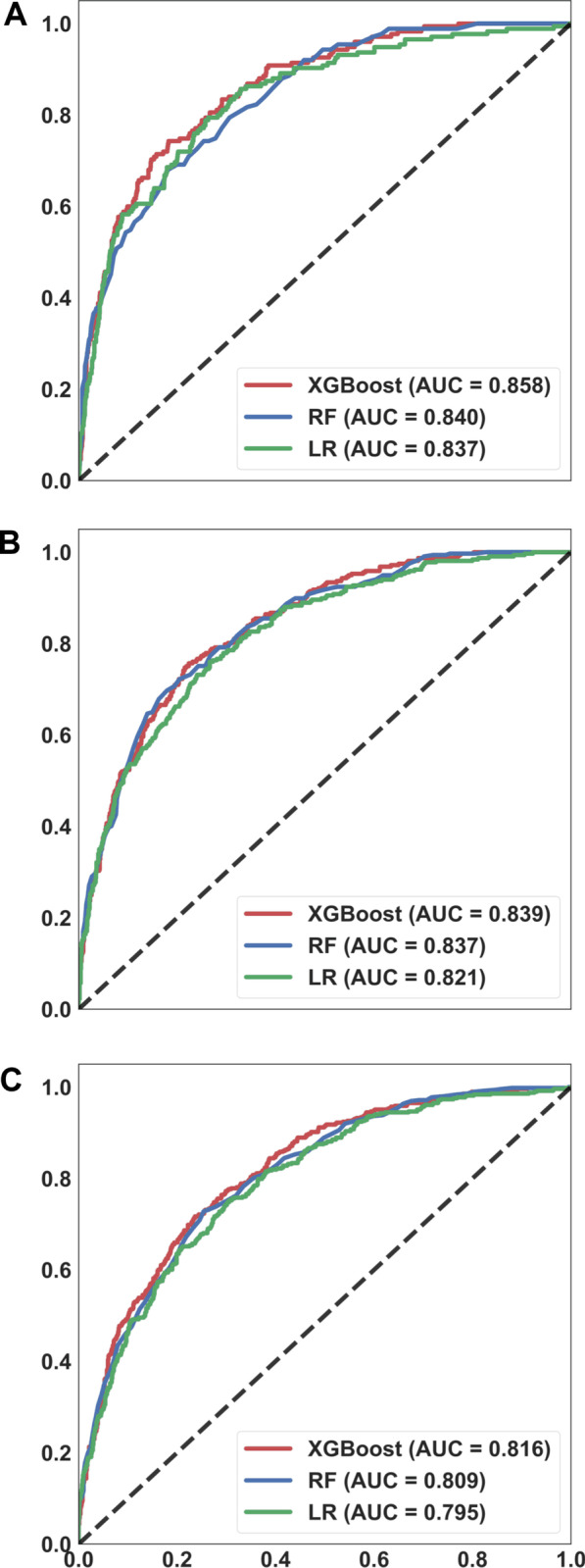
Receiver operating characteristic curves demonstrating the performance of the three machine learning models for predicting the mortality at 30-day (**A**), 90-day (**B**), and 1-year (**C**). Area under curve (**A** 30-day, XGBoost 0.858, 95% CI 0.830–0.886; RF 0.840, 95% CI 0.811–0.869; LR 0.837, 95% CI 0.805–0.869) (**B** 90-day, XGBoost 0.839, 95% CI 0.816–0.863; RF 0.837, 95% CI 0.813–0.861; LR 0.821, 95% CI 0.795–0.847) (**C** 365-day, XGBoost 0.816, 95% CI 0.786–0.832; RF 0.809, 95% CI 0.786–0.832; LR 0.795, 95% CI 0.771–0.819)

We compared the performance among the three ML models to predict the 30-day, 90-day, and 1-year mortality. The XGBoost tended to have the highest accuracy, with the AUC value of using XGBoost to predict 30-day, 90-day and 1-year mortality were 0.858, 0.839 and 0.816, respectively (Fig. 2) (see Additional file 1: Table S1 for the detailed metric of the performance). The calibration curve showed fair consistency between the predicted values and the actual observed values (Additional file 1: Fig. S2). The decision curve analysis also demonstrated the good overall net benefits within a relatively wide range of threshold probabilities (Additional file 1: Fig. S3). Given that the accuracy in predicting mortality appeared to be similar among the three ML models, we used DeLong's test to determine the difference of AUCs among the three ML models and found that XGBoost had slightly better accuracy in predicting mortality than those in LR and RF (30-day mortality XGBoost vs. RF *p* < 0.01, XGBoost vs. LR *p* = 0.06; 90-day mortality XGBoost vs. RF *p* = 0.70, XGBoost vs. LR *p* = 0.04; 1-year mortality, XGBoost vs. RF *p* = 0.20, XGBoost vs. LR *p* < 0.01).

### Explanation of the model at the domain and feature level

**Fig. 3:**
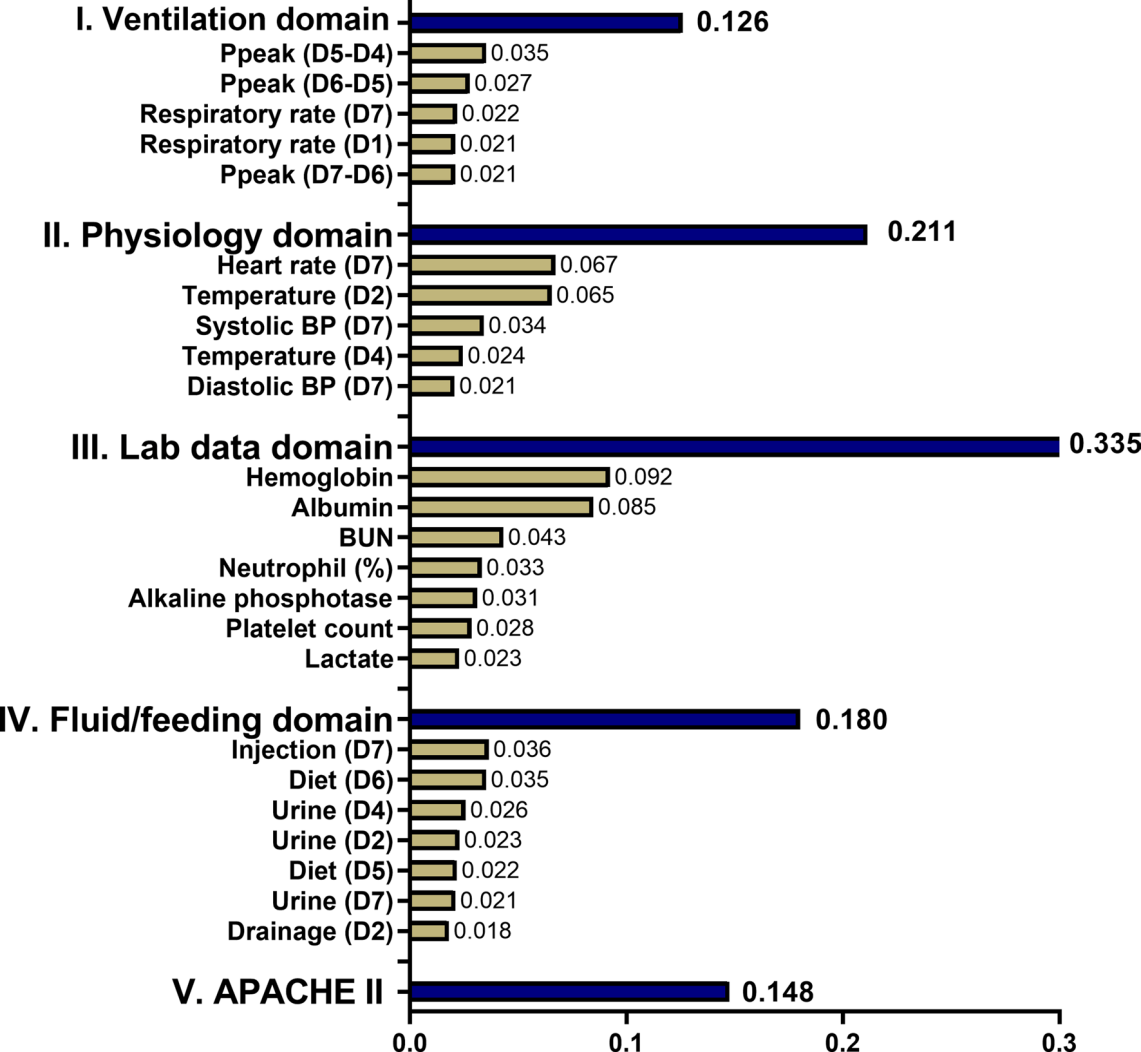
Cumulative relative feature importance of top 25 features categorised by main clinical domains in predicting 1-year mortality

**Fig. 4:**
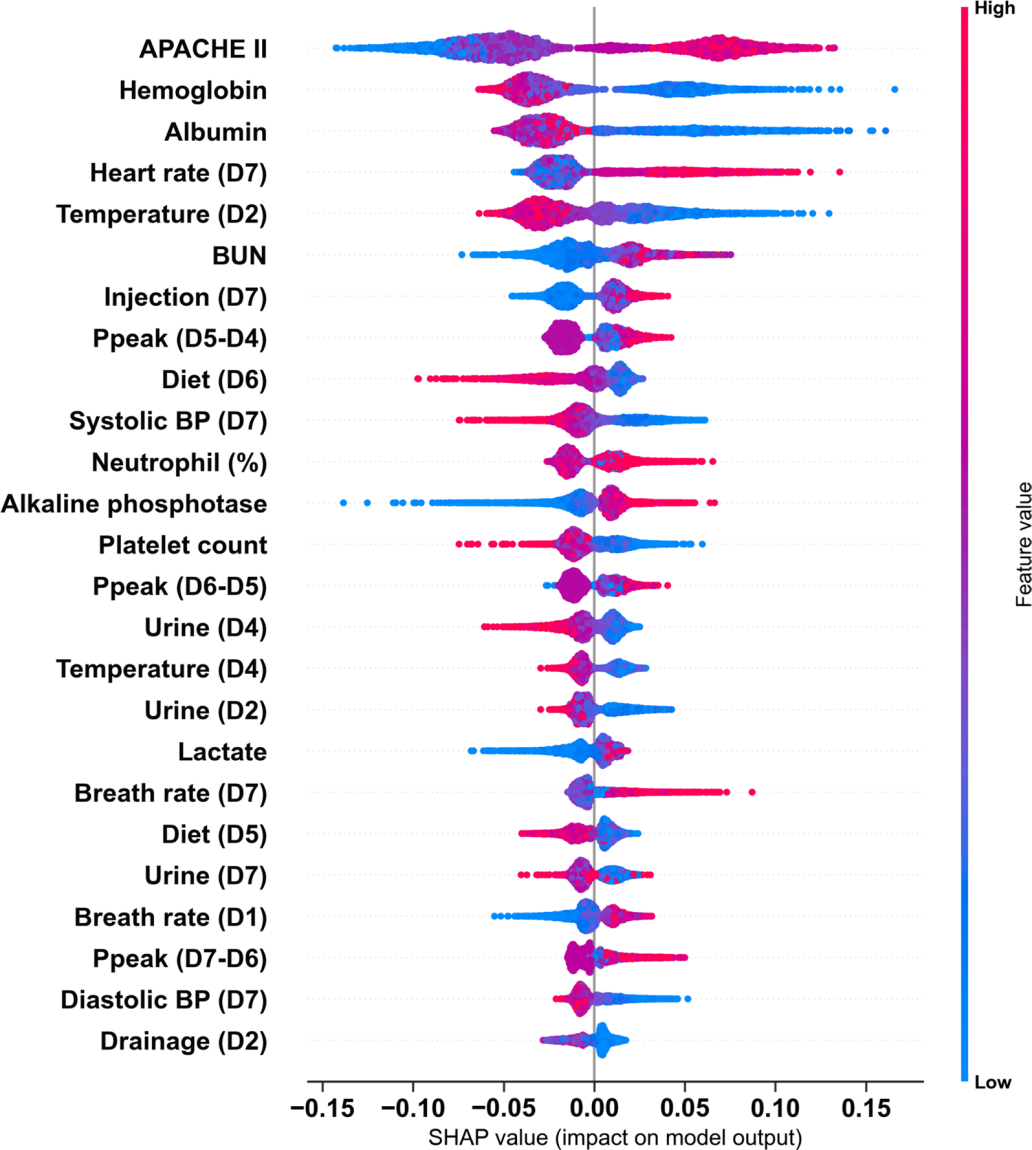
SHAP to illustrate the 1-year mortality prediction model at feature level. SHapley Additive exPlanation (SHAP)

**Fig. 5:**
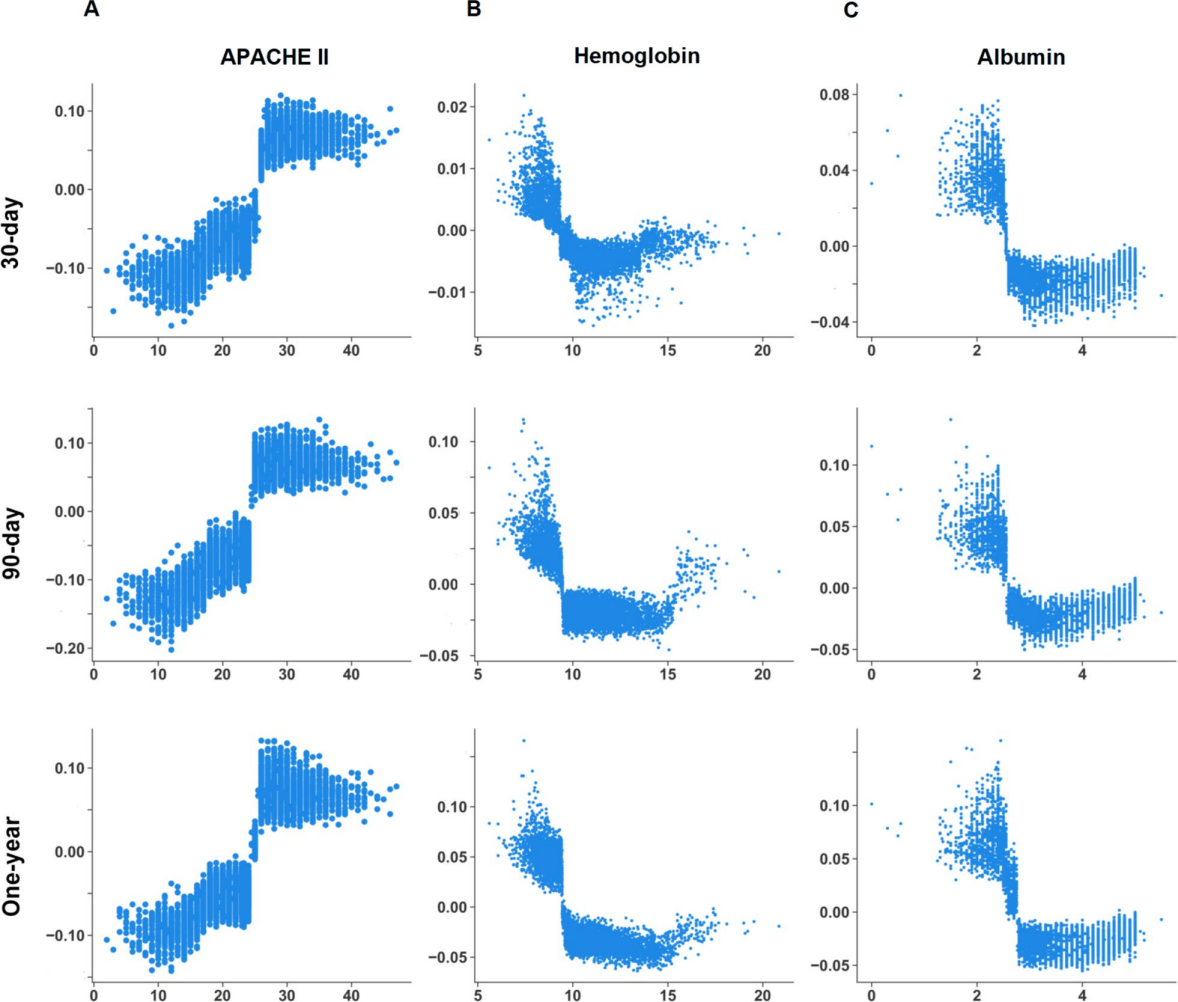
Partial dependence plot by SHAP value in predicting 1-year mortality with distinct time points. APACHE II score (**A**), haemoglobin (**B**), and albumin (**C**)

We illustrated the ML model at the clinical-domain level, feature level, and individual level. With respect to domain-level explanation in the 1-year mortality prediction model, we categorised the top 25 features by main clinical domains in accordance with clinical workflow for management among critically ill ventilated patients (Fig. 3). In the 1-year mortality prediction model, the cumulative feature importance of the ventilatory domain, fluid domain, physiologic domain, laboratory data domain, and APACHE II was 0.126, 0.211, 0.335, 0.180, and 0.147, respectively (Additional file 1: Fig. S4 and Additional file 1: Fig. S5 for 30-day and 90-day mortality prediction model). To further illustrate the model at the feature level, we employed SHAP summary plot to demonstrate how these features affect the probability of mortality (Fig. 4). Using SHAP summary plot, both the strength and direction of each feature were explicitly illustrated. For example, a higher APACHE II score was associated with a higher probability of 1-year mortality, whereas a higher level of hemoglobulin and albumin was inversely associated with mortality (Fig. 4 for 1-year mortality prediction model; Additional file 1: Fig. S6 and Additional file 1: Fig. S7 for 30-day and 90-day mortality prediction model). To further elucidate how each feature affects the probability of mortality in the ML model, we used PDP plot of the three crucial features, including APACHE II score, level of hemoglobulin and albumin. As shown in Fig. 5, APACHE II score higher than approximately 25, level of hemoglobulin lower than nearly 9 mg/dL, level of albumin lower than 2.5–3 mg/dL were consistently associated with an increased risk of mortality at the three time points (Fig. 5). Collectively, these visualised interpretations at domain and feature level based on clinical workflow in critical care should provide intuitive explanations of the ML model to the physician.

### Explanation of the ML model at the individual level

**Fig. 6:**
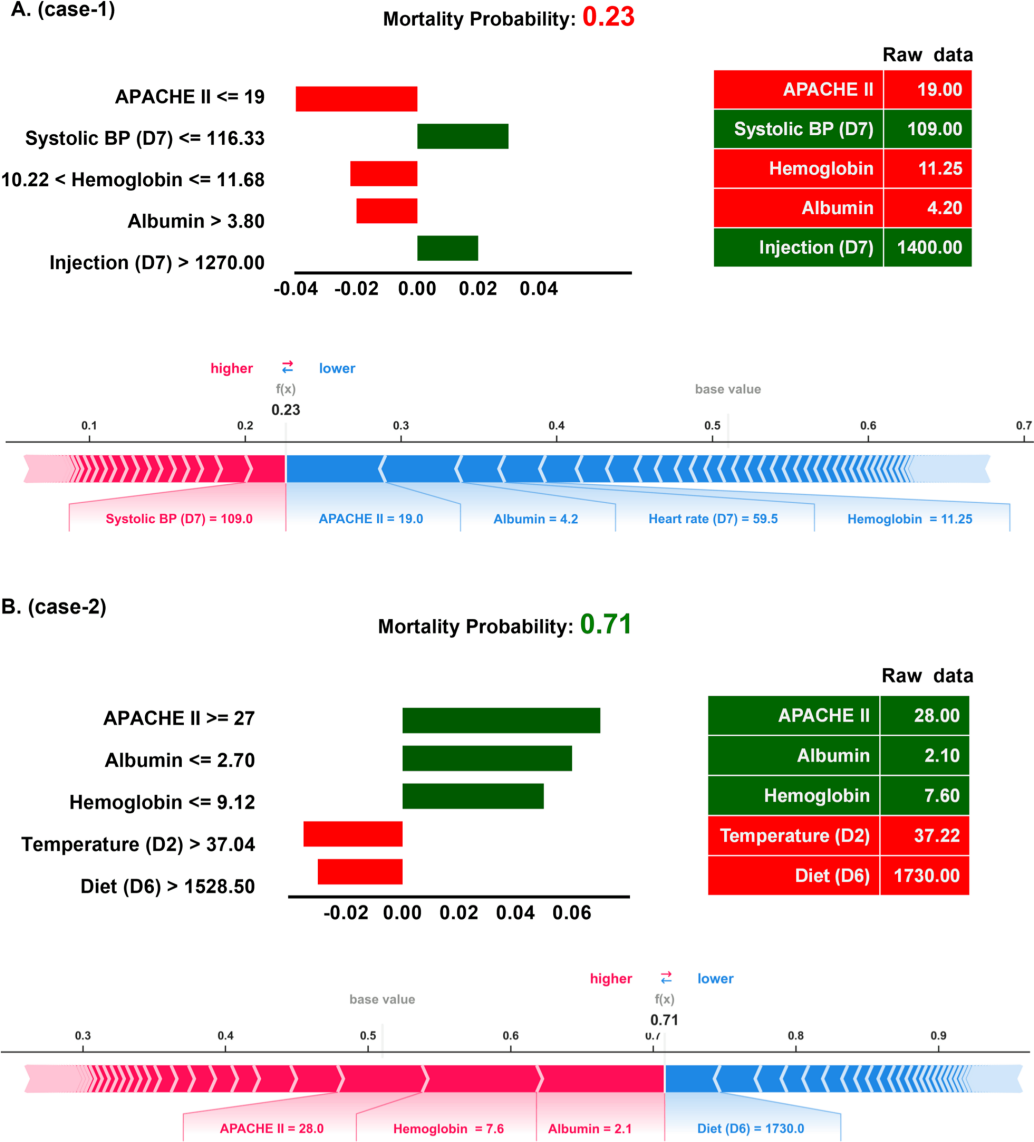
Local interpretable model-agnostic explanations (LIME) and SHAP force plots of two representative individuals. SHapley Additive exPlanation (SHAP)

We next used LIME and SHAP force plots of crucial features to illustrate the overall impact of key features on the 1-year mortality prediction model in individual patients. As shown in Fig. 6, the overall predicted probability of mortality, incremental mortality effects of variables (green), and decremental mortality effects of variables (red) of two representative patients were illustrated in the LIME plot (Fig. 6). For example, in case-1, the predicted probability for 1-year mortality was relatively low (0.23) due to a number of decremental conditions, consisting of a low APACHE II score (19) and a high albumin (4.2 mg/dL) as well haemoglobin (11.25 mg/dL), although a slightly low systolic blood pressure on day-7 (109 mmHg) and high injected fluid on day-7 (1400 mL). The SHAP force plot illustrated similar findings of key features, including APACHE II score, albumin, haemoglobin, and systolic blood pressure on day-7 (Fig. 6A). In contrast, the probability of 1-year mortality in case-2 was apparently high (0.71) due to a number of incremental conditions, including a high APACHE II (28) and low albumin (2.1 mg/dL) as well as haemoglobin (7.6 mg/dL), despite a relatively normal blood temperature on day-2 (37.2 Celsius) and feeding diet amount on day-6 (1730 mL). SHAP force plot showed similar findings with a succinct summary of the real data, whereas the cut-point of each features omitted (Fig. 6B). Taken together, these explanations at the individual level were in line with the explanation at the feature level and should be able to mitigate the black-box concern in the application in the field of medical AI.

## Discussion

In the present study, we used an explainable ML approach to establish a long-term outcome prediction model in critically ill ventilated patients. We mainly employed XGBoost to establish the mortality prediction model with high accuracy and illustrated distinct determinants among models to predict short- and long-term mortality. We further used the SHAP summary plot and PDP for the post-hoc interpretation of the model at the feature level. Additionally, we used LIME and SHAP force plots to illustrate the prediction model at the individual level. These data demonstrated the application of a real-world dataset and explainable ML approach to establish a physician understandable long-term mortality prediction model in critically ill ventilated patients. These findings are crucial for early risk stratification and intervention, such as the implementation of physical, nutritional and psychological support, in high-risk patients.

AI is increasingly employed in critical care medicine with a growing number of registered clinical trials [19, 20], and the majority of studies aimed to predict the outcome, mainly short-term mortality, in critically ill patients [21]. Pirracchio et al. using a composite ML-based mortality prediction model, so-called Super ICU Learner Algorithm, among 24,508 patients in Medical Information Mart for Intensive Care (MIMIC) II database, reported the accuracy to predict hospital mortality was 0.85 (95% CI: 0.84–0.85), whereas the accuracy of SOFA, a widely used conventional scoring system, was merely 0.71 (95% CI: 0.71–0.72) [22]. Similarly, Kong et al. focusing on 16,688 septic patients in the MIMIC III database, reported the accuracy to predict hospital mortality of the least absolute shrinkage and selection operator (LASSO), RF, gradient boosting machine (GBM) and LR were 0.829, 0.829, 0.845 and 0.833, respectively [10]. Shillan et al. recently summarised a total of 47 studies using ML models to predict hospital mortality in critically ill patients and found that the average AUC was nearly 0.83 among studies of 1000–10,000 patients, and the finding is consistent with our data that the AUC to predict 30-day mortality was approximately 0.85 using data from approximately 7000 patients (Fig. 3). Taken together, these evidence showed the similar accuracy of using ML models to predict short-term mortality and highlight the needs for predicting long-term mortality in critically ill patients.

The long-term outcome is currently an emerging research issue in critically ill patients given the much improved short-term outcome with the advance of critical care medicine in the past two decades [2, 23]. A number of studies including our previous studies focusing on critically ill cancer patients and surgical patients have identified factors associated with long-term mortality in critically ill patients using a conventional statistical approach [4, 5, 24]. In line with our study, García-Gallo et al*.*, linking 5650 patients with sepsis in the MIMIC-III database with the Social Security Administration Death Master File to obtain the Out-of-hospital mortality dates, recently reported that the accuracy of predicting 1-year mortality through using day-1 data and the Stochastic Gradient Boosting (SGB) method was approximately 0.80 [25]. Due to the comprehensive day-1 data is essential in aforementioned study focusing on using day-1 data, nearly 54% (8186/15,254) of critically ill septic patients were excluded due to incomplete day-1 data, and the proposed model tended to predict the acute mortality given that merely 269 patients with hospital-stay shorter than 24 h were excluded [25]. In the present study, we used the week-1 data including comprehensive ventilatory data to predict mortality after week-1; therefore, the dataset enables us to predict short-, medium- and long-term outcome with interpretability among critically ill ventilated patients. Unlike a wide span of study year in MIMIC III (2001–2012), the dataset during 2015–2019 as we used in the present study appears to reflect current management and prognosis in critical care medicine. However, both our study and the study performed by García-Gallo et al. were single centre study, and prospective multi-centre studies are required to validate our findings.

In the present study, we provided interpretability at the domain and feature level. We found that the cumulative feature importance of the ventilatory domain decreased along with the prediction window, and the finding was consistent with the clinical condition that ventilatory condition mainly reflects acute/short-term outcome, instead of long-term outcome (Fig. 3). Similarly, among features within the laboratory domain, acute parameters, including lactate level and platelet count, were associated with short-term mortality, whereas subacute parameters, including the level of haemoglobin and albumin, were associated with long-term outcome (Figs. 3, 5). Indeed, low albumin has been found to be associated with mortality in critically ill patients [26–28]. Recent studies on COVID-19 infection also revealed that higher baseline albumin was associated with lower severity of COVID-19 infection and fewer adverse outcomes, including the requirement of ICU admission, development of acute respiratory distress syndrome, incident venous thromboembolism, and readmissions within 90 days [29, 30]. In line with our finding that anaemia appears to be a crucial determinant for 1-year mortality, Waner et al. recently conducted a population-based study to address the prevalence and recovery from anaemia among 6901 critically ill patients [5]. Waner et al. found that the prevalence of anaemia at 3, 6 and 12 months post hospitalisation was 56%, 52% and 45%, respectively. A higher hospital discharge haemoglobin concentration was independently associated with decreased 1-year mortality (adjHR, 0.95 per 1-g/dL increase; 95% CI, 0.90–0.99) [5]. Collectively, we used explainable machine learning to illustrate the key determinants for 1-year mortality in ML models, and the identified determinants are consistent with clinical evidence in critical care medicine.

Although AI technologies have been widely applied in numerous fields, but adoption of AI algorithms with the black-box issue in the critical care field remains uncommon given that intensivist may not take action without realising the rationale behind the suggested decision [19]. Indeed, interpretability might not be required in the decision support system in a low-risk environment given that an error does not lead to serious consequences; however, interpretability is substantially required in decision with high stake for certain AI applications, including in critical care medicine, criminal justice, and business judgment [7]. The goal of an explainable AI (XAI) system is to make a decision similar to human behaviours by providing explanations [31]; therefore, the explanation in medical XAI should be in line with the workflow of the physician as the main clinical domains we have shown in the present study (Fig. 3). Notably, to directly open the black box is somehow difficult, and the currently proposed measures for explanation mainly analysed the model after training, so-called post-hoc interpretability [7, 31]. In the present study, we focused on the interpretability of ML model among individuals whose certainty of prediction was high, including patients with a high/low probability of mortality. In contrast, the explanation among individuals with an ambiguous probability of mortality could be irrational given that the prediction itself is uncertain. Additionally, the interpretability focused on the local explanation of key features instead of global model interpretability with a detailed explanation of parameters and weights within the model [32]. Given that the aforementioned explanation of key features is independent of the underlying AI model, and this model-agnostic interpretation method is hence increasingly employed among distinct ML models, particularly tree-based models [33, 34]. In addition to explanations of the entire model and individual features using SHAP plots as we have shown in the present study (Figs. 4, 5), more studies are warranted to explore feature interaction effects [34]. Collectively, the goal of XAI in critical care medicine is to provide an intensivist-understandable and model-agnostic explanation of the model, but not a comprehensive elucidation of the AI algorithm.

There are limitations in this study. First, this study is a single-centre study, and further validation study is warranted to confirm our findings. However, the overall short- and long-term mortality rate is consistent with previous studies (Additional file 1: Fig. S8. survive curve of the enrolled subjects) [35]. Moreover, the data were retrieved from the dataset in a real-world setting; the concern with respect to generalisation should be largely mitigated. Second, the technology readiness level (TRL) of the present study might merely be TRL-4 [36], but we believe that TRL-5 can be achieved through integrating an optimal user interface given that the data in the present study were retrieved from structured EMR of real-world practice. Third, the observation nature of the present study, and the causal inference should be taken with caution. Therefore, the future practical application of the present study should tend to be a computer-aided triage. Fourth, the interpretability of the model tends to be a descriptive metric instead of a causal inference explanation. Additionally, the single imputation method by the average value could lead to a bias in this study.

## Conclusions

We established a critical care dataset with long-term outcome through linking a real-world critical care dataset with the death registry file of a nationwide database. We used three ML models, including XGBoost, RF and LR, to predict 30-day, 90-day and 1-year mortality. Furthermore, we employed clinical domain-based cumulative feature importance, SHAP plot as well as PDP plots for visualised interpretation at the feature level and SHAP/LIME plot to illustrate key determinants at the individual level. We think these explainable ML approaches should largely mitigate the issue of black-box. Future prospective multi-centre studies are warranted for the validation and landing of the proposed model.

## Supplementary Information

**Additional file 1. Supplemental table 1**. Metrics of performance of the three machine learning models to predict 30-day, 90-day and one-year mortality.** Supplemental Figure 1**. Flow diagram of the analytic pipeline in the study.** Supplemental Figure 2**. Calibration curves of the three machine learning models for predicting the mortality at 30-day (A), 90-day (B), and one-year (C).** Supplemental Figure 3**. Decision curve analyses of the three machine learning models to predict the mortality at 30-day (A), 90-day (B), and one-year (C).** Supplemental Figure 4**. Cumulative relative feature importance of top 25 features categorised by main clinical domains in predicting 30-day mortality.** Supplemental Figure 5**. Cumulative relative feature importance of top 25 features categorised by main clinical domains in predicting 90-day mortality.** Supplemental Figure 6**. SHAP to illustrate the 30-day mortality prediction model in feature level. Abbreviation: SHapley Additive exPlanation (SHAP).** Supplemental Figure 7**. SHAP to illustrate the 90-day mortality prediction model in feature level. Abbreviation: SHapley Additive exPlanation (SHAP).** Supplemental Figure 8**. Survival curve of the enrolled critically ill ventilated patients.

## Data Availability

All of the data and materials are provided in the manuscript and the Additional data. The code has been put in public Github, and is available via https://github.com/GitTerrySu/Predict-long-term-mortality.
