# Supplementary material for: Explainable machine learning to predict long-term mortality in critically ill ventilated patients: a retrospective study in central Taiwan
Source: BMC Med Inform Decis Mak. 2022 Mar 25;22:75. doi: 10.1186/s12911-022-01817-6 (PMC8953968; doi:10.1186/s12911-022-01817-6)
Supplement: Supplementary file 1 — Additional file 1. Supplemental table 1. Metrics of performance of the three machine learning models to predict 30-day, 90-day and one-year mortality. Supplemental Figure 1. Flow diagram of the analytic pipeline in the study. Supplemental Figure 2. Calibration curves of the three machine learning models for predicting the mortality at 30-day (A), 90-day (B), and one-year (C). Supplemental Figure 3. Decision curve analyses of the three machine learning models to predict the mortality at 30-day (A), 90-day (B), and one-year (C). Supplemental Figure 4. Cumulative relative feature importance of top 25 features categorised by main clinical domains in predicting 30-day mortality. Supplemental Figure 5. Cumulative relative feature importance of top 25 features categorised by main clinical domains in predicting 90-day mortality. Supplemental Figure 6. SHAP to illustrate the 30-day mortality prediction model in feature level. Abbreviation: SHapley Additive exPlanation (SHAP). Supplemental Figure 7. SHAP to illustrate the 90-day mortality prediction model in feature level. Abbreviation: SHapley Additive exPlanation (SHAP). Supplemental Figure 8. Survival curve of the enrolled critically ill ventilated patients. [file 12911_2022_1817_MOESM1_ESM.pdf]

Supplemental table 1. Metrics of performance of the three machine learning models to predict 30-day, 90-day and one-year mortality

		Precision	Sensitivity	F1-Score	Specificity	Brier-Score	Accuracy[a]
**XGBOOST**	30-day	0.96	0.75	0.87	0.80	0.137	0.798
	90-day	0.91	0.74	0.84	0.77	0.159	0.764
	One-year	0.84	0.74	0.78	0.73	0.176	0.732
**RF**	30-day	0.96	0.74	0.85	0.77	0.162	0.767
	90-day	0.91	0.75	0.82	0.74	0.171	0.743
	One-year	0.84	0.73	0.78	0.73	0.184	0.726
**LR**	30-day	0.95	0.73	0.87	0.79	0.155	0.785
	90-day	0.91	0.73	0.84	0.77	0.169	0.763
	One-year	0.83	0.71	0.78	0.73	0.184	0.723

[a] (TP+TN) / (TP+FN+TN+FP). Abbreviation: Extreme gradient boosting, XGBoost; random forest, RF; logistical regression, LR.

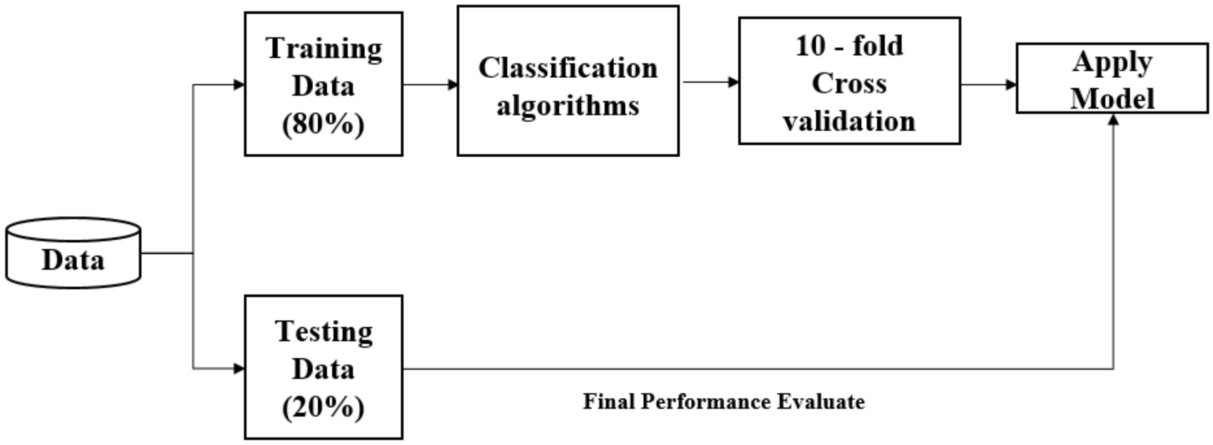

Supplemental Figure 1. Flow diagram of the analytic pipeline in the study

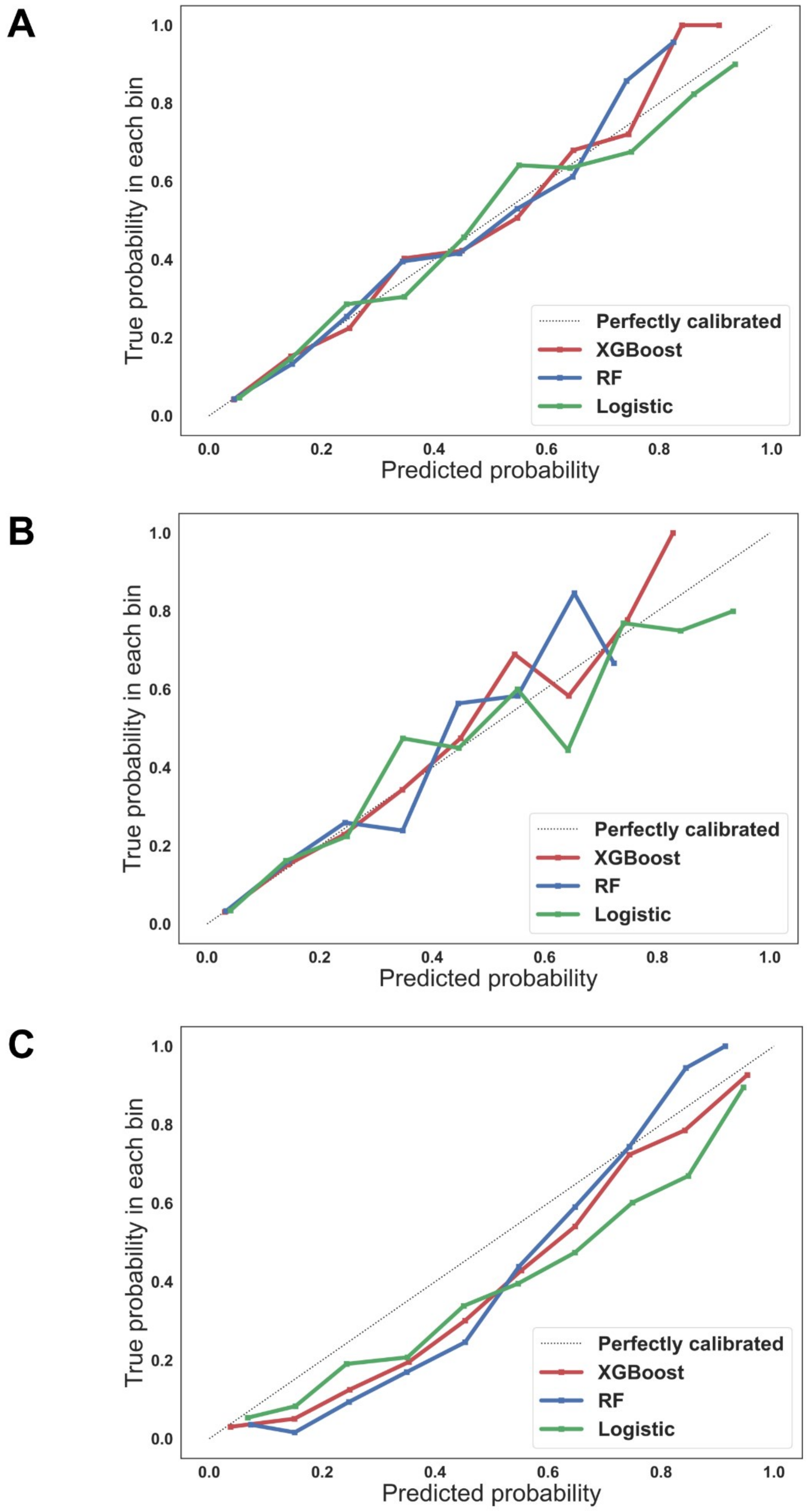

Supplemental Figure 2. Calibration curves of the three machine learning models for predicting the mortality at 30-day (A), 90-day (B), and one-year (C).

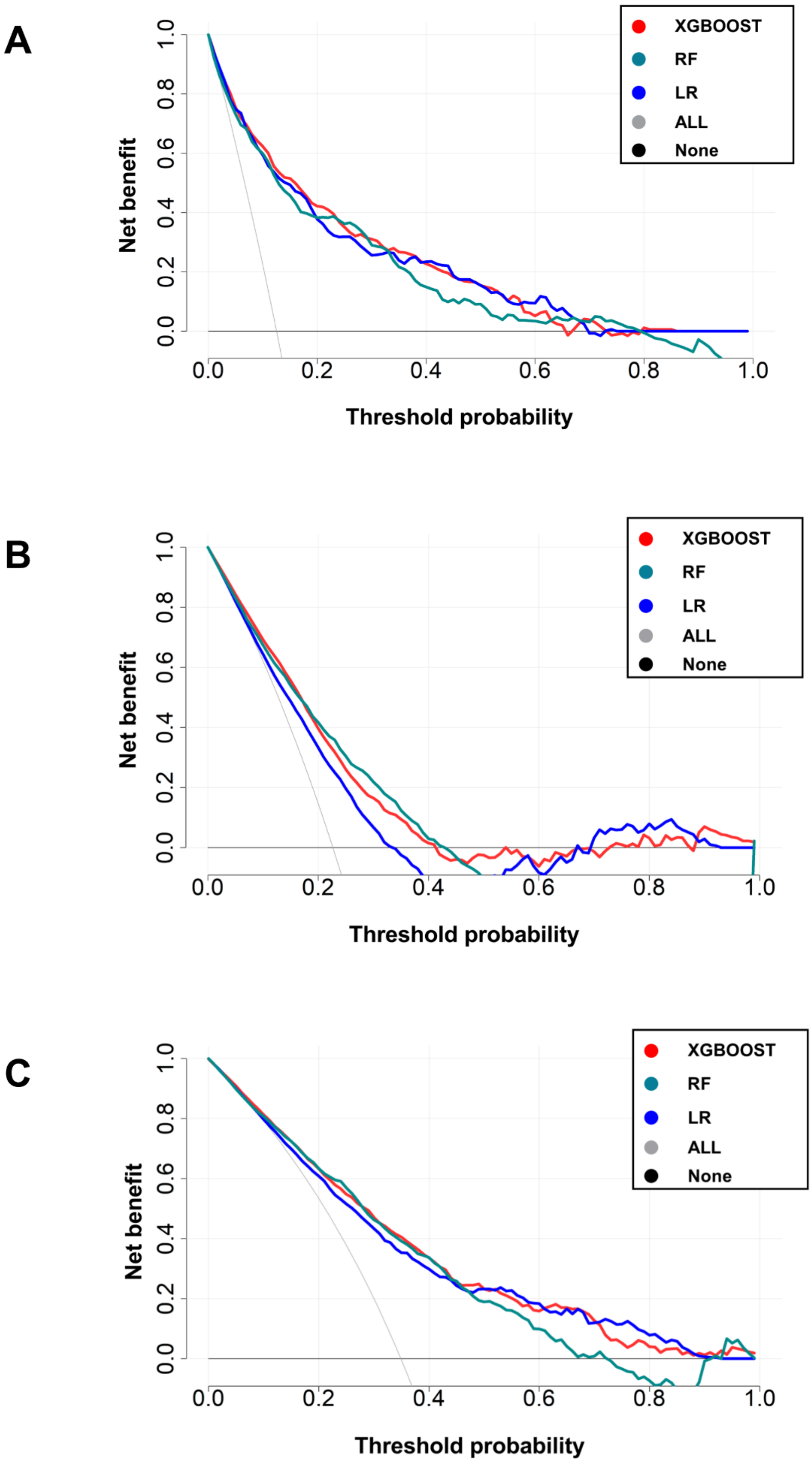

Supplemental Figure 3. Decision curve analyses of the three machine learning models to predict the mortality at 30-day (A), 90-day (B), and one-year (C).

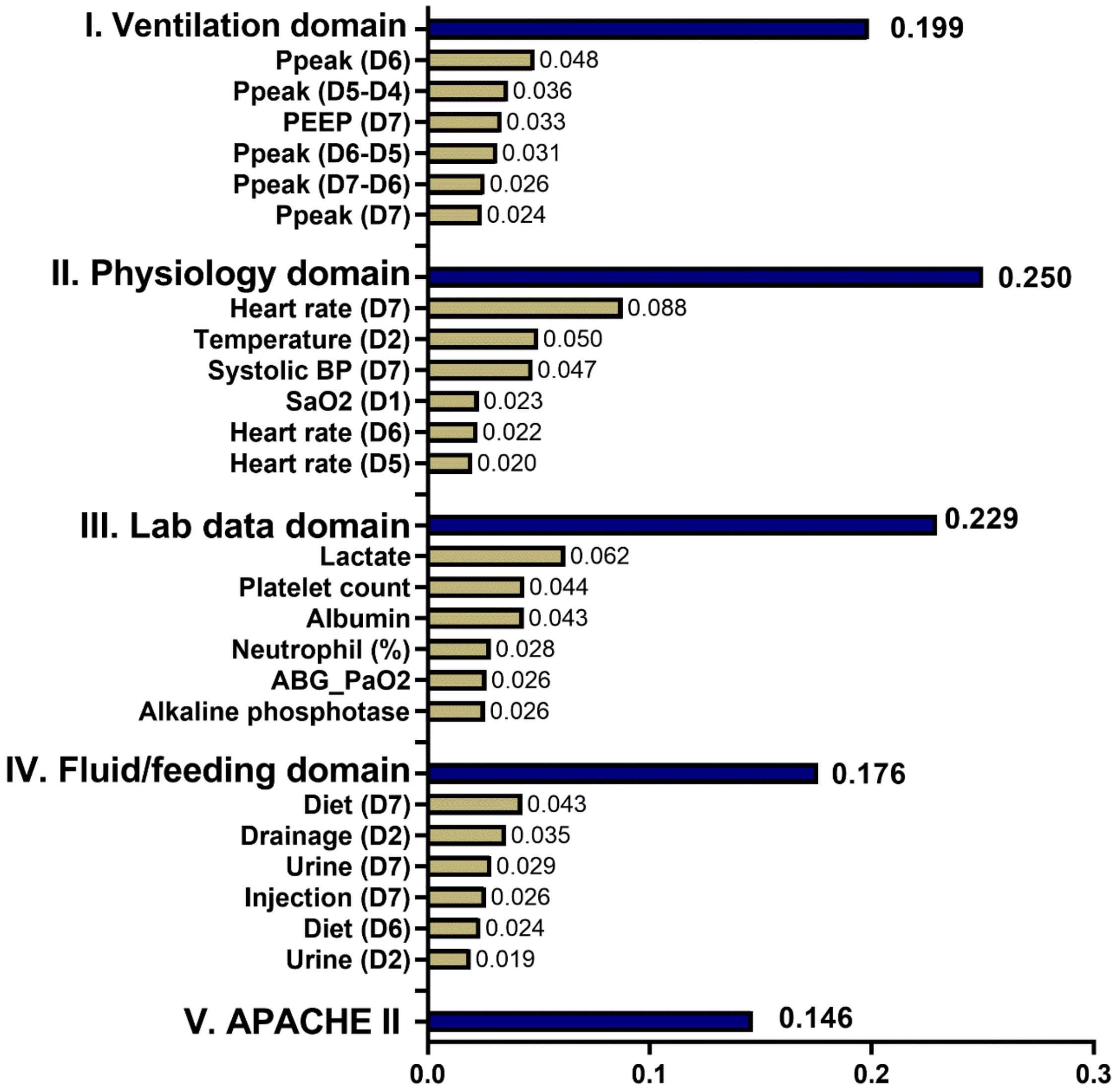

Supplemental Figure 4. Cumulative relative feature importance of top 25 features categorised by main clinical domains in predicting 30-day mortality

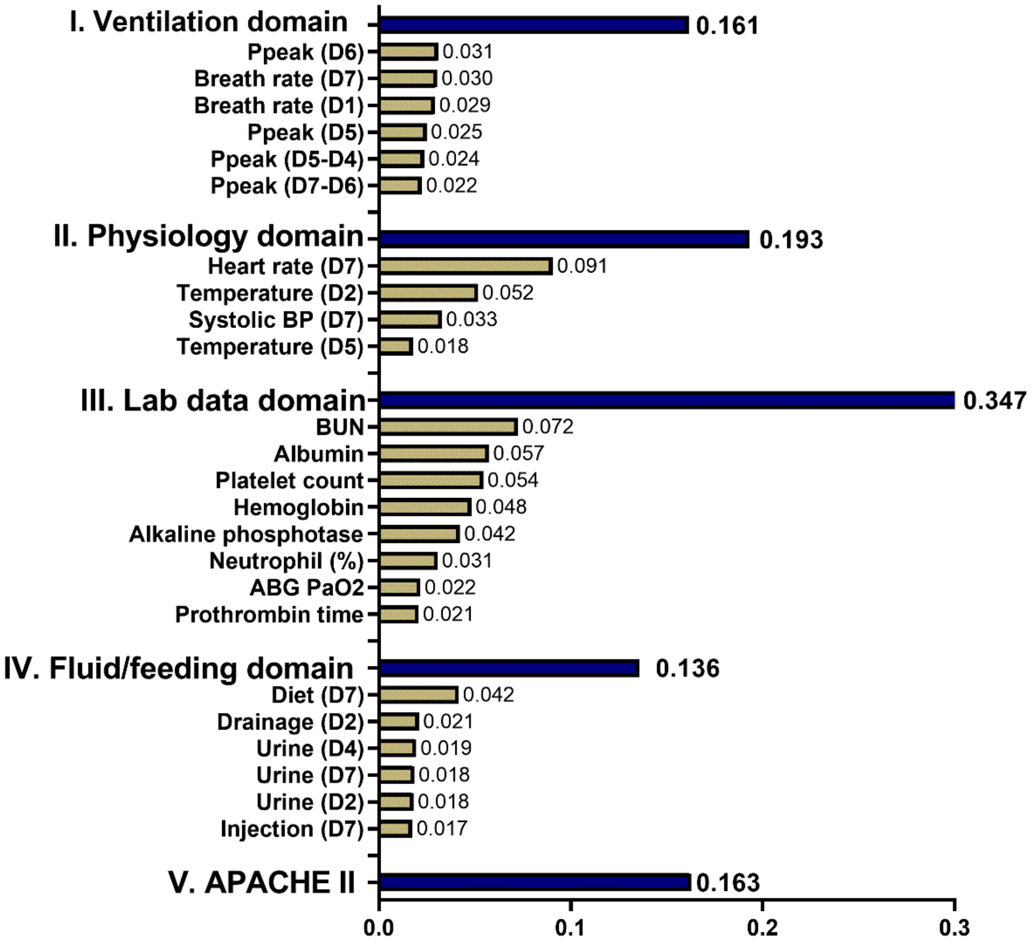

Supplemental Figure 5. Cumulative relative feature importance of top 25 features categorised by main clinical domains in predicting 90-day mortality

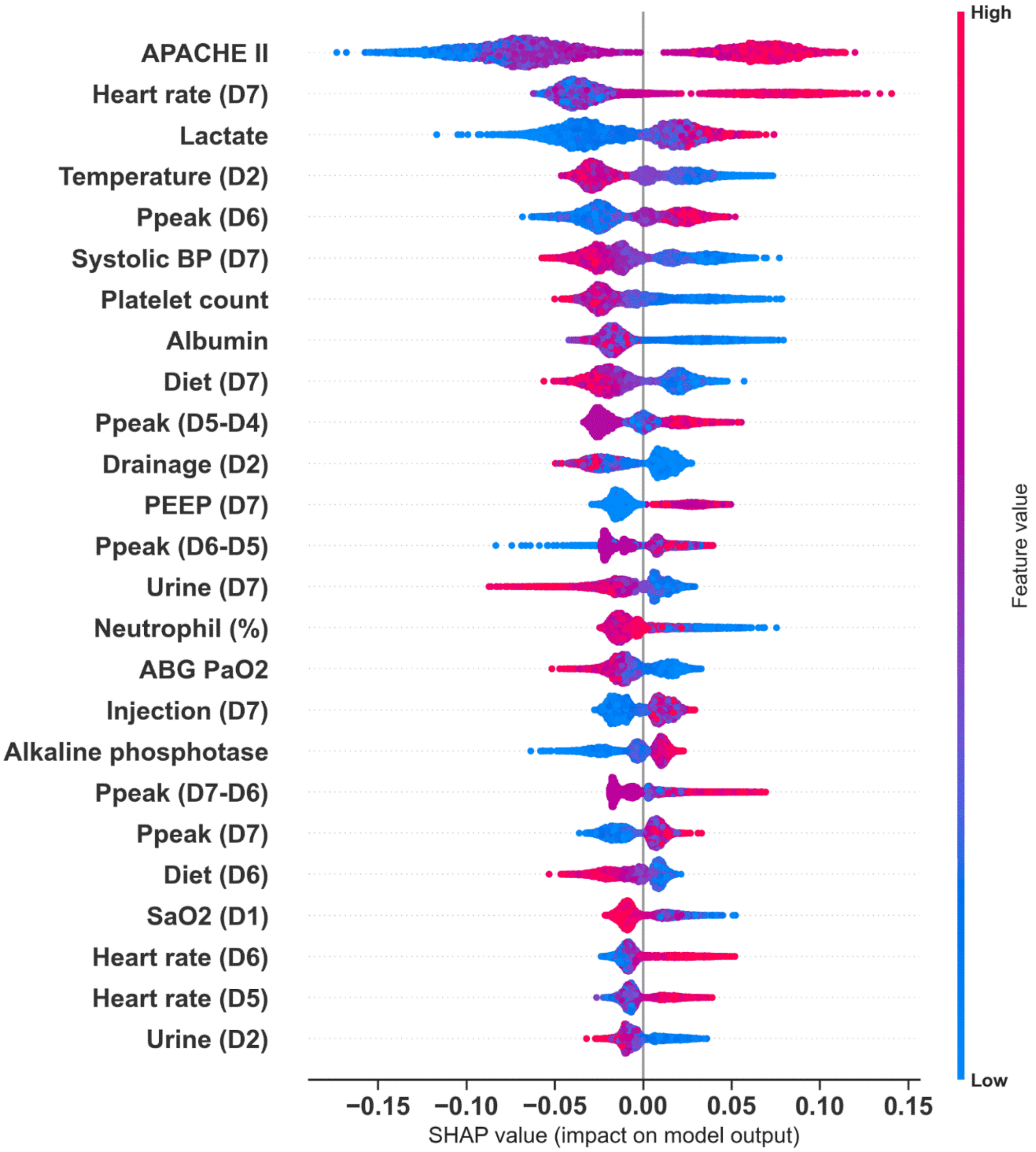

Supplemental Figure 6. SHAP to illustrate the 30-day mortality prediction model in feature level. Abbreviation: SHapley Additive exPlanation (SHAP)

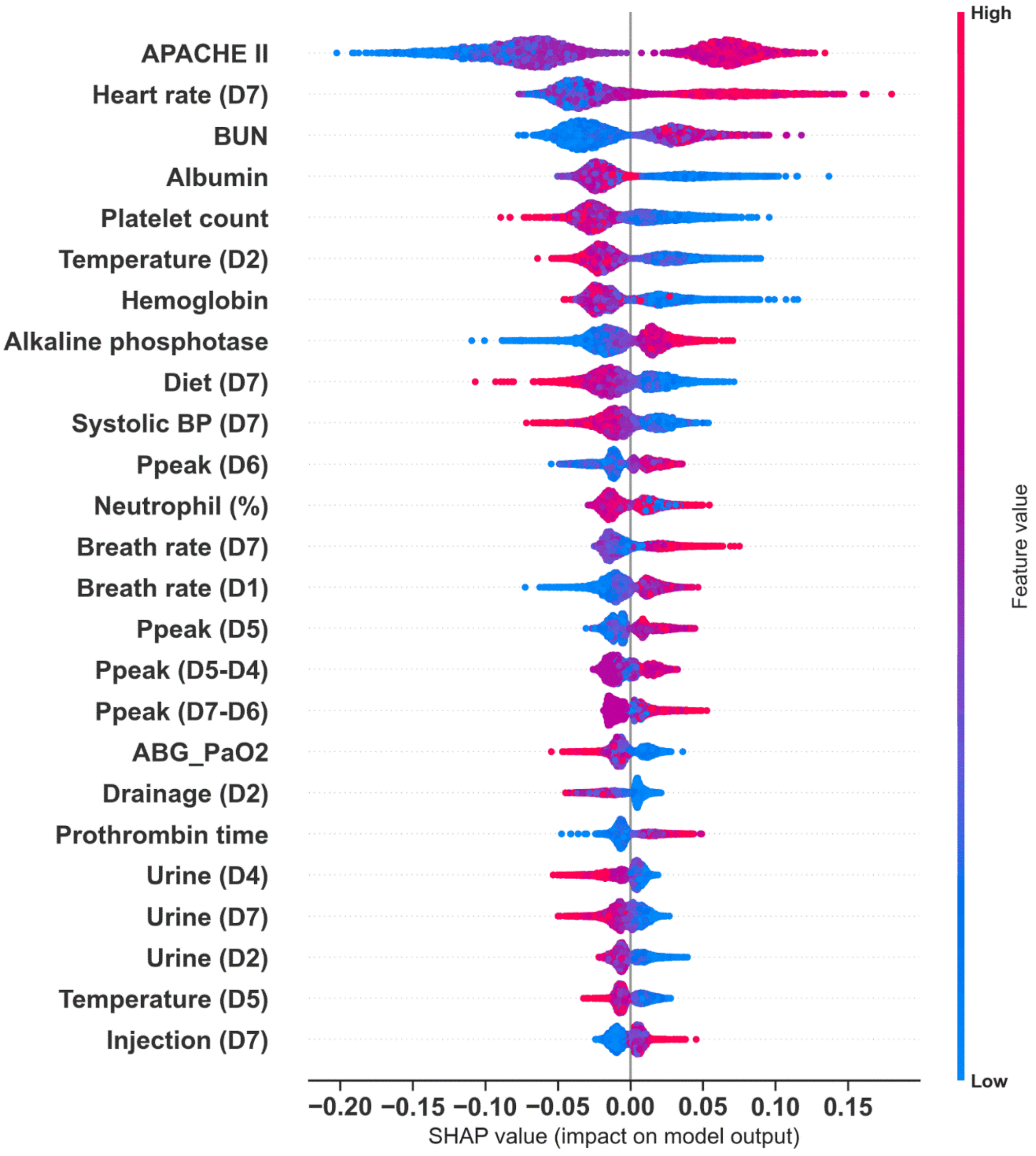

Supplemental Figure 7. SHAP to illustrate the 90-day mortality prediction model in feature level. Abbreviation: SHapley Additive exPlanation (SHAP)

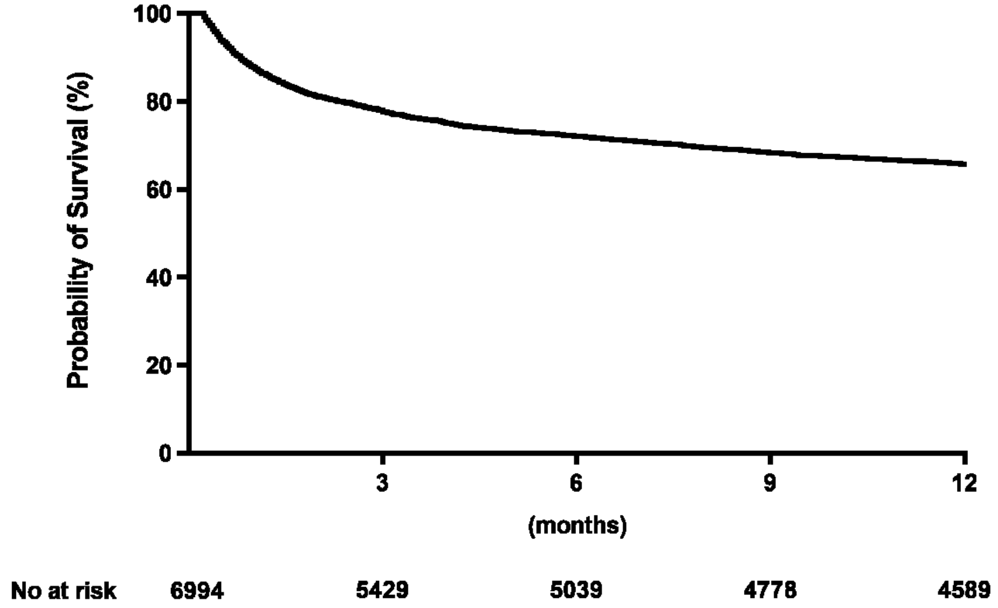

Supplemental Figure 8. Survival curve of the enrolled critically ill ventilated patients
